# Artificial Intelligence–Based Computerized Digit Vigilance Test in Community-Dwelling Older Adults: Development and Validation Study

**DOI:** 10.2196/73038

**Published:** 2025-11-26

**Authors:** Gong-Hong Lin, Dorothy Bai, Yi-Jing Huang, Shih-Chieh Lee, Mai Thi Thuy Vu, Tsu-Hsien Chiu

**Affiliations:** 1 International Ph.D. Program in Gerontology and Long-Term Care, College of Nursing, Taipei Medical University, Taipei, Taiwan; 2 School of Gerontology and Long-Term Care, College of Nursing, Taipei Medical University, 250 Wuxing Street, Xinyi District, Taipei, Taiwan, 886 2-2736-1661 ext 6332, 886 2-2377-2842; 3 School of Occupational Therapy, College of Medicine, National Taiwan University, Taipei, Taiwan; 4 Department of Physical Medicine and Rehabilitation, National Taiwan University Hospital, Taipei, Taiwan; 5 Department of Psychiatry, National Taiwan University Hospital, Taipei, Taiwan; 6 Nam Dinh University of Nursing, Nam Dinh, Nam Định Province, Vietnam; 7 Department of Civil Engineering, National Taiwan University, Taipei, Taiwan

**Keywords:** artificial intelligence, assessment, cognition, age-friendly, attention

## Abstract

**Background:**

The Computerized Digit Vigilance Test (CDVT) is a well-established measure of sustained attention. However, the CDVT only measures the total reaction time and response accuracy and fails to capture other crucial attentional features such as the eye blink rate, yawns, head movements, and eye movements. Omitting such features might provide an incomplete representative picture of sustained attention.

**Objective:**

This study aimed to develop an artificial intelligence (AI)–based Computerized Digit Vigilance Test (AI-CDVT) for older adults.

**Methods:**

Participants were assessed by the CDVT with video recordings capturing their head and face. The Montreal Cognitive Assessment (MoCA), Stroop Color Word Test (SCW), and Color Trails Test (CTT) were also administered. The AI-CDVT was developed in three steps: (1) retrieving attentional features using OpenFace AI software (CMU MultiComp Lab), (2) establishing an AI-based scoring model with the Extreme Gradient Boosting regressor, and (3) assessing the AI-CDVT’s validity by Pearson *r* values and test-retest reliability by intraclass correlation coefficients (ICCs).

**Results:**

In total, 153 participants were included. Pearson *r* values of the AI-CDVT with the MoCA were −0.42, −0.31 with the SCW, and 0.46–0.61 with the CTT. The ICC of the AI-CDVT was 0.78.

**Conclusions:**

We developed an AI-CDVT, which leveraged AI to extract attentional features from video recordings and integrated them to generate a comprehensive attention score. Our findings demonstrated good validity and test-retest reliability for the AI-CDVT, suggesting its potential as a reliable and valid tool for assessing sustained attention in older adults.

## Introduction

Sustained attention can be defined as a state of readiness to detect and respond to certain changes in the environment that occur at random intervals over extended periods of time [1,2]. The effects of aging on sustained attention are complex; while some aspects of attention may decline, some studies suggested that older adults can maintain more stable performance on certain vigilance tasks than their younger counterparts [3,4]. This phenomenon is not thought to reflect general superiority but rather several factors, including that older adults report fewer task-unrelated thoughts (ie, less mind-wandering) [5,6] and adopt a more cautious, top-down response strategy that prioritizes accuracy over speed. Research indicated that the performance and variability of sustained attention in older adults are related to frailty and fall risks [7,8]. Consequently, monitoring sustained attention in older adults could be an effective way to manage the health of older adults.

The Computerized Digit Vigilance Test (CDVT) is a widely used measure of sustained attention with established reliability and validity [9,10]. However, like many traditional cognitive tests, it relies on a single modality of data, performance metrics (ie, reaction time and accuracy). This unimodal approach overlooks a rich stream of behavioral data that contains valuable information about a person’s attentional state. For instance, subtle increases in the blink duration, downward gaze shifts, and slight head drooping are all well-documented physical manifestations of waning vigilance and attentional lapses [11-14]. By failing to capture these overt behaviors, the test’s reliability is constrained, as fluctuations in reaction time alone might not fully or consistently reflect an individual’s true attentional state.

The concept of using automated, vision-based systems to infer cognitive states from behavioral cues is well-established in other fields. For instance, in transportation safety, extensive research has focused on developing systems that monitor driver vigilance by analyzing features like the blink rate, gaze direction, and head pose to detect drowsiness and prevent accidents [15,16]. Similarly, in educational technology and human-computer interactions, computer vision techniques are used to assess student engagement and cognitive load by tracking similar behavioral markers [17,18]. These applications demonstrate the value of using objective, observable behaviors as proxies for internal attentional states. However, despite success in these domains, this multimodal approach has seen limited application in enhancing standardized clinical neuropsychological assessments, particularly for older adults.

Artificial intelligence (AI) offers a promising approach to analyze facial features [19,20], potentially providing valuable supplementary data for attention assessments [11]. AI-powered software can directly extract attentional features, such as the eye blink rate, yawn frequency, head rotation, and eye movements, from images or videos of faces [19,20]. Traditionally, collecting these features required specialized equipment such as eye trackers or virtual reality headsets [11]. AI-based software offers a more cost-effective and feasible alternative for collecting attentional data [19,20], particularly in clinical settings and for older adult populations.

In this study, we attempted to bridge this gap by leveraging AI to integrate these disparate data streams. AI, particularly machine learning models, is exceptionally well-suited for this task because it can learn complex, nonlinear patterns from multimodal data, automatically determining the optimal weight to assign each feature—from reaction time to eyelid distance—to produce a single, comprehensive score. Therefore, in this study, we attempted to enhance the psychometric properties of the CDVT by integrating an additional modality of data. We developed an artificial intelligence–based Computerized Digit Vigilance Test (AI-CDVT) that uses machine learning to combine traditional performance metrics with facial and behavioral features captured on video. The primary hypothesis was that by creating a more comprehensive, multimodal assessment, we could improve the test-retest reliability of the measure while maintaining its convergent validity.

## Methods

### Participants

Participants were recruited via convenience sampling from community care centers in Taiwan. A member of the research team visited these centers, provided an oral presentation to groups of older adults explaining the study’s purpose and procedures, and invited interested individuals to enroll. Participants were eligible if they met the following criteria: (1) aged 65 years or older, (2) having had no hospitalization in the past 6 months, and (3) willing to participate in the study. Exclusion criteria were a doctor-diagnosed disability or an unwillingness to record videos during the CDVT assessment.

### Procedures

This study consisted of 2 waves of data collection. In the first wave, participants were assessed once to gather cross-sectional data. Assessments included the CDVT and Montreal Cognitive Assessment (MoCA) [21]. In the second wave, participants were assessed twice, with a 2-week interval between assessments, to collect test-retest data. Both assessments involved the CDVT and MoCA. Additionally, in the first assessment, the Stroop Color Word Test (SCW) [22] and both parts of the Color Trails Test (CTT) [23] were assessed. At the start of the first assessment session for each participant, a trained assessor administered a brief questionnaire to collect demographic information, including age, sex, and educational attainment (Multimedia Appendix 1). Each participant was individually assessed by a trained assessor in a quiet, one-on-one setting at a community care center. All assessments were conducted on a laptop computer with a 15.6-inch screen, an Intel i5 processor, 8 GB of DDR4 RAM, and a GTX 950 graphics card. Participants were seated approximately 50 cm from the screen. Video recordings capturing participants’ heads and faces were obtained using the laptop’s built-in HD webcam (1.3 megapixels) during CDVT assessments.

### Measures

#### Computerized Digit Vigilance Test (CDVT)

**Figure 1.**
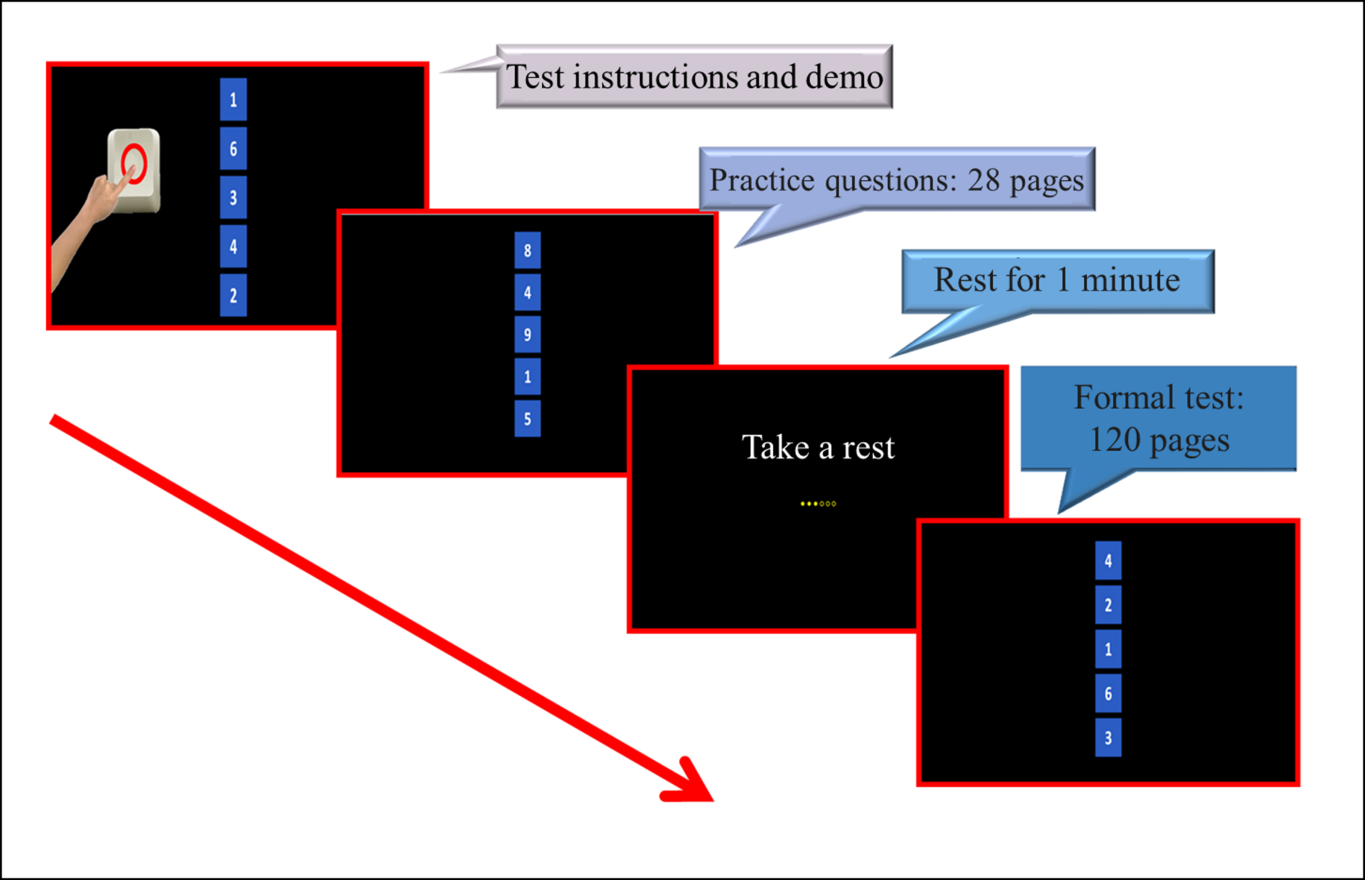
Computerized Digit Vigilance Test process and items.

The CDVT is a computer-based test designed to assess sustained attention. Participants use 2 buttons (a circle and an X) to respond to the presence of the numeral “6” on the screen (Figure 1). The test records response times and errors to evaluate sustained attention, with shorter times indicating better focus. Studies showed good validity and reliability of the CDVT in patients with stroke and schizophrenia [9,10].

#### Montreal Cognitive Assessment (MoCA)

The MoCA is a cognitive screening tool used to assess various cognitive domains in older adults. The MoCA evaluates short-term memory, visuospatial abilities, executive functions, attention, concentration, working memory, language, and orientation. The total score on the MoCA is 30, with a higher score indicating better cognitive performance. The MoCA demonstrated good validity and reliability in older adults [24,25]. Further details on the instrument and its authorized use are provided in Multimedia Appendix 1.

#### Stroop Color Word Test (SCW)

The SCW is a neuropsychological assessment tool used to evaluate cognitive function, particularly attention and executive functioning. During the SCW, participants are presented with a list of color words printed in incongruent font colors (eg, “RED” printed in blue). Participants are asked to verbally name the color of the font, inhibiting the prepotent response of reading the word itself. Faster completion times on the SCW are indicative of better attention and executive functioning. Good reliability and validity of the SCW were reported in older adults [26]. The specific version used in this study is cited in Multimedia Appendix 1.

#### Color Trails Test (CTT)

The CTT is a language-free version of the Trail Making Test, designed to measure sustained attention and divided attention in adults. The CTT involves connecting circles in an ascending numbered sequence (from 1 to 25) in the CTT1 and alternating between pink and yellow colors while connecting numbers in ascending order in the CTT2. Numbers are presented twice, once in pink and once in yellow, requiring the client to consecutively follow the sequence while avoiding the same color in a row. The time taken to complete each part of the CTT is recorded in seconds. Good reliability of the CTT was determined in older adults [27]. The specific version used in this study is cited in Multimedia Appendix 1.

### Data Analysis

To determine whether the AI-CDVT can evaluate participants’ attention according to their facial videos and CDVT output, we developed the AI-CDVT in 3 steps, retrieving attentional features, establishing an AI-based scoring model, and validating the AI-CDVT. In the first step, we adopted OpenFace (CMU MultiComp Lab) [28] to retrieve attentional features from a participant’s face in videos. Attentional features were extracted from each video frame. The eight primary features included: (1-2) the distance between the eyelids for each eye, serving as a continuous measure of eye openness (a smaller distance indicates greater closure, as a proxy for blinks); (3) the distance between the lips, indicating mouth opening or yawning; (4‐6) head rotation angles across 3 axes, corresponding to pitch (nodding), yaw (side-to-side rotation), and roll (ear-to-shoulder tilt); and (7-8) the X-Y coordinates of the estimated gaze point. For each of the 120 trials in the CDVT, we aggregated the frame-by-frame data by calculating the mean value for each of these 8 features. Within any given trial, video frames where OpenFace failed to successfully track facial features were excluded from this calculation; the mean was computed only from the successfully captured frames. Furthermore, because the CDVT is a computerized test that requires a response before proceeding to the next item, there were no missing test items. This process yielded a single value per feature for each trial. Finally, we calculated the overall mean and SD of these 120 trial-level values for each feature. These 16 summary statistics (8 features×2 statistics), along with the mean and SD of the reaction time and response accuracy from the original CDVT, constituted the final set of 20 input features for the AI model. All input features were normalized to a common scale before being used in the model.

In the second step, we adopted an AI algorithm, the Extreme Gradient Boosting (XGBoost) regressor [29], to receive inputs of attentional features and estimate scores of the CDVT as scores of the AI-CDVT. The XGBoost regressor leverages the aggregation of random forest regressors to improve the predictive accuracy and has been successfully used in medication situations [29]. In addition, the XGBoost regressor offers an importance ranking of attentional features to generate AI-CDVT scores.

In the third step, we validated the AI-CDVT using a 3-fold cross-validation procedure [30]. In this procedure, we randomly separated our data into 3 subdatasets. Then, we validated the AI-CDVT 3 times. For each validation instance, we used 2 subdatasets to train the XGBoost regressor and used the remaining subdataset to validate the convergent validity and test-retest reliability. Notably, a different subdataset was used each time for validation.

Then, we calculated the mean and SD of the indices of validity across the 3 validation times. The convergent validity was examined using Pearson *r* values to evaluate associations between AI-CDVT scores and those of the MoCA, CTT, and SCW. In addition, we also calculated Pearson *r* values between CDVT scores and those of the MoCA, CTT, and SCW. If Pearson *r* values of the AI-CDVT were similar to those of the CDVT, the convergent validity of the AI-CDV was considered good [31].

The test-retest reliability was examined using an intraclass correlation coefficient (ICC) to evaluate the agreement of scores in the test and retest assessment sections. An ICC value of > 0.75 indicates good test-retest reliability [32].

Finally, to provide a more rigorous evaluation of the AI-CDVT’s clinical utility beyond correlational metrics, we conducted a supplementary classification analysis. The goal was to determine whether the multimodal features captured by the AI-CDVT offered incremental value in predicting a clinically relevant outcome. We used the MoCA score as a proxy for the cognitive status, dichotomizing participants into 2 groups based on the common clinical cutoff for potential cognitive impairment “at risk” (MoCA score ≤25) and “not at risk” (MoCA score >25). We then compared the performance of the 2 XGBoost classification models in predicting this binary outcome. The first model (“CDVT-Only”) used only the mean reaction time and mean accuracy from the CDVT as input features. The second model (“AI-CDVT Top 5”) used the 5 most important features identified in our initial regression model (see Table 1), which included a mix of performance and facial metrics. Specifically, these top 5 features were the mean reaction time, the mean distance between the right eyelids, the mean Y coordinate of the gaze point, the mean accuracy, and the mean angle of head rotation in the Y axis (yaw). Model performance was evaluated using the accuracy and *F*_1_-score across the same 3-fold cross-validation procedure.

**Table 1.** Rank of importance of attentional features in the artificial intelligence–based Computerized Digit Vigilance Test.

Rank	Attentional feature
1	Mean of the reaction time
2	Mean of the distance between the right eyelids
3	Mean of the Y coordinate of the gaze point
4	Mean of the accuracy
5	Mean of the angle of head rotation in the Y axis (yaw)
6	SD of the Y coordinate of the gaze point
7	SD of the distance between the right eyelids
8	Mean of the distance between the left eyelids
9	SD of the angle of head rotation in the Z-axis (roll)
10	SD of the X coordinate of the gaze point
11	Mean of the angle of head rotation in the Z-axis (roll)
12	Mean of the distance between the lips
13	Mean of the angle of head rotation in the X-axis (pitch)
14	SD of the accuracy
15	SD of the distance between the lips
16	SD of the angle of head rotation in the X-axis (pitch)
17	SD of the distance between the left eyelids
18	Mean of the X coordinate of the gaze point
19	SD of the angle of head rotation in the Y-axis (yaw)
20	SD of the reaction time

### Ethical Considerations

This study was approved by an institutional review board (Taipei Medical University, approval number: N202010008), and all participants provided written informed consent. Participants received a small honorarium of a gift voucher equivalent to US $3 for their time and participation. The informed consent process explicitly detailed the nature of the video recording and its purpose. To ensure data privacy and confidentiality, all video data were stored on an encrypted, offline hard drive. The raw video files were permanently deleted immediately after the deidentified facial features were extracted by OpenFace software, and only these numerical, nonidentifiable data points were retained for analysis.

## Results

In total, 153 participants were used for the cross-sectional data (n=87) and test-retest data (n=66). In the cross-sectional data, the average age of participants was 70.8 years, and most of them were female (64/87, 73.6%). In general, they had no cognitive impairment according to the average MoCA score (25.5, SD 2.8). Characteristics of the test-retest data were similar to those of cross-sectional data, as there were no significant differences between them. Table 2 shows further information on these characteristics.

**Table 2.** Demographic and baseline cognitive characteristics of study participants.

Variable	Cross-sectional data (n=87)	Test-retest data (n=66)	*t *test or chi-square (*df*)	*P* value
Age (years), mean (SD)	70.8 (5.9)	72.4 (5.9)	1.66 (151)	.10
Sex (male), n (%)	64 (73.6)	49 (74.2)	.009 (1)	.92
Educational level, n (%)			6.06 (4)	.19
Graduate school	6 (6.9)	5 (7.6)		
University	37 (42.5)	17 (25.8)		
High school	32 (36.8)	36 (54.5)		
Junior high school	6 (6.9)	3 (4.5)		
Elementary school	6 (6.9)	5 (7.6)		
MoCA^a^, mean (SD)	25.5 (2.8)	25 (3.3)	1.01 (151)	.31
CDVT^b^, mean (SD)	239.6 (25.2)	247.3 (26.8)	1.82 (151)	.07
AI-CDVT^c^, mean (SD)	239.2 (24.6)	247.3 (27)	1.93 (151)	.06

a MoCA: Montreal Cognitive Assessment.

b CDVT: Computerized Digit Vigilance Test.

c AI-CDVT: artificial intelligence–based Computerized Digit Vigilance Test.

Pearson *r* values of AI-CDVT scores with external criteria were −0.42 (SD 0.19) with the MoCA score, −0.31 (SD 0.16) with the SCW score, 0.46 (SD 0.17) with CTT1 score, and 0.61 with CTT2 scores, and. Pearson *r* values of the AI-CDVT were similar to those of the CDVT. Specifically, Pearson *r* values between the CDVT score and external criteria scores were −0.41 (SD 0.17), −0.29 (SD 0.10), 0.44 (SD 0.21), and 0.55 (SD 0.15), respectively.

The average ICC of the AI-CDVT was 0.78 with a range of 0.68‐0.84 according to 3-fold cross-validation (Table 3). Similar results were found for the CDVT, for which the average ICC was 0.71 with a range of 0.64‐0.76.

**Table 3.** Test-retest reliability of the artificial intelligence–based Computerized Digit Vigilance Test (AI-CDVT) and original Computerized Digit Vigilance Test (CDVT) over a 2-week interval.

Performance metric	CDVT	AI-CDVT
Cross-validation: Fold 1, ICC^a^ (95% CI)	0.72 (0.44‐0.87)	0.84 (0.67‐0.93)
Cross validation: Fold 2, ICC^a^ (95% CI)	0.76 (0.5‐0.89)	0.81 (0.61‐0.92)
Cross validation: Fold 3, ICC^a^ (95% CI)	0.64 (0.32‐0.83)	0.68 (0.27‐0.87)
Average across 3-fold cross validation, mean (SD)	0.71 (0.06)	0.78 (0.09)

a ICC: intraclass correlation coefficient.

In the supplementary classification analysis, the model using the top 5 AI-CDVT features demonstrated improved performance in predicting the cognitive status (MoCA ≤25) compared to the model using only traditional CDVT metrics. Specifically, the AI-CDVT Top 5 model achieved an average accuracy of 58.9% and an *F*_1_-score of 50.6% across the 3 folds. This represents a modest improvement over the CDVT-only model, which scored an accuracy of 57.5% and an *F*_1_-score of 48.6%.

Importance values of attentional features of the AI-CDVT are listed in Table 1. The top 5 most important attentional features were the mean of the reaction time, the distance between the right eyelids, the Y coordinate of the gaze point, mean of the accuracy, and the horizontal rotation angle of the head (Table 1).

## Discussion

### Principal Findings

The primary rationale for developing the AI-CDVT was to determine if integrating multiple, objective behavioral data streams could enhance the psychometric robustness of a standard sustained-attention test. While the AI-CDVT did not shorten the test’s administration time nor demonstrate superior correlations with external cognitive measures, our findings support the study’s main hypothesis: the AI-CDVT achieved a notable improvement in the test-retest reliability while maintaining convergent validity comparable to the original CDVT. This suggests that by capturing a richer, multimodal snapshot of an individual’s attentional state, the AI-CDVT offers a more stable and reliable assessment tool.

Compared to the CDVT, the AI-CDVT demonstrated equivalent validity and improved test-retest reliability. This enhanced reliability might be attributed to the incorporation of additional attentional features in the AI-CDVT [33,34]. By incorporating a broader range of attentional features, AI-CDVT scores likely benefited from greater stability and robustness. The AI-CDVT score may be less vulnerable to fluctuations solely due to response speed or accuracy. The findings suggest that incorporating a wider range of attentional features during assessments can lead to more reliable scores.

While the AI-CDVT’s convergent validity correlations were similar to those of the original CDVT, its primary clinical and functional value lies not in superior predictive accuracy but in its enhanced psychometric robustness and the potential for a more nuanced interpretation of attentional performance. The improved test-retest reliability, for example, is a direct clinical benefit, as it provides a more stable and trustworthy score for longitudinal monitoring or evaluating intervention effects [32]. Furthermore, by capturing a wider array of behaviors like gaze shifts and eyelid closure, the AI-CDVT provides a richer dataset. This could allow clinicians to move beyond a single performance score to understand the underlying nature of an individual’s attentional difficulties, distinguishing, for instance, between general cognitive slowing and specific lapses in vigilance [33]. This multimodal approach offers a more holistic view of sustained attention [34], paving the way for future research into distinct behavioral phenotypes of attentional decline that are invisible to traditional, response-time-based measures.

The results of our supplementary classification analysis warrant careful interpretation. The modest improvements (1.4% in accuracy and 2.0% in *F*_1_-score), while not dramatic, provide a crucial proof-of-concept. They suggest that the multimodal features captured by the AI-CDVT contain a small but detectable signal that is relevant to clinical outcomes (ie, potential cognitive impairment as flagged by the MoCA). This finding lends support to our central hypothesis that integrating objective behavioral markers, even if their individual predictive power is small, can incrementally enhance the clinical utility of a traditional cognitive test. While the immediate clinical impact of this gain is limited, it establishes a methodological foundation for future work. It is plausible that this incremental value could be magnified in larger, more clinically diverse samples or by using more advanced machine learning architectures, highlighting a promising avenue for subsequent research.

It is important to appropriately position the contribution of this study. We did not develop a new AI algorithm from the ground up; rather, our innovation lies in the practical application and integration of established, open-source tools (OpenFace and XGBoost) to enhance a standard clinical assessment. The novelty of this work is therefore not in a deep methodological invention but in demonstrating the feasibility and clinical utility of creating a multimodal assessment of sustained attention. By showing that combining behavioral response data with easily captured facial metrics can improve psychometric properties like reliability, this study provides a proof-of-concept and a methodological template for other researchers aiming to enrich traditional neuropsychological tests with objective, behavioral data streams.

### Comparison to Prior Work

By leveraging the flexibility of AI models, such as XGBoost regressors, the AI-CDVT can potentially address the challenge of interpreting individual attentional features in isolation [35]. For instance, directly comparing attention between individuals with high accuracy but slow response times and those with lower accuracy but faster responses can be difficult [36]. The AI-CDVT illustrates a viable method for integrating and interpreting various attentional features into a unified score for future studies and attentional tests.

A related methodological consideration is the selection of the 20 input features. This set was not chosen arbitrarily. Each feature was included because it had been identified in the existing scientific literature as a physiological or behavioral marker related to attention and vigilance [11,12,37]. Our approach was to build a comprehensive model based on these established, theory-driven features. While this could introduce redundancy between some inputs (eg, left and right eyelid distance), the chosen XGBoost algorithm is robust to such multicollinearity, as it inherently performs feature selection during its training process [38]. The final feature importance rankings in Table 1 are a direct result of this process, demonstrating how the model itself identified the most valuable contributors from the initial set of theory-driven features.

An examination of the feature importance rankings (Table 1) provides insights into how the AI-CDVT achieves its robust performance. While traditional metrics like reaction time and accuracy are unsurprisingly the most critical predictors, the model also heavily weighs physiological and behavioral markers. For example, the “distance between the eyelids” emerged as a top feature. This is consistent with literature linking decreased eyelid aperture and blink rate dynamics to drowsiness and lapses in vigilance [39]. Similarly, the “Y coordinate of the gaze point” was highly predictive, likely because a downward shift in gaze is a well-established behavioral marker of task disengagement and mind-wandering [40,41]. The inclusion of these features allows the AI-CDVT to capture subtle, moment-to-moment fluctuations in attentional states that are not reflected in response times alone, thereby providing a more comprehensive and ecologically valid assessment.

A final consideration is the interpretability of the AI model, which is a critical factor for its clinical adoption [42,43]. Complex models like XGBoost are often termed “black boxes” because they do not produce a simple, transparent formula in the way a linear regression model can [44]. There is an inherent tradeoff between the high predictive accuracy of such models and their direct interpretability [45]. In this study, our primary tool for interpretation is the feature importance table (Table 1). While it does not explain how the features are combined for any single individual, it provides clinicians with a clear and valuable understanding of what the model is paying attention to [46]. It confirms that the AI-CDVT’s score is driven by a combination of performance metrics and behavioral patterns (eg, eye closure and gaze aversion) that are clinically consistent with inattention. This allows clinicians to trust that the model’s logic aligns with established knowledge, even if the precise weighting algorithm remains complex [47,48].

### Limitations

This study has several notable limitations. First, the generalizability of our findings is constrained by the characteristics of our sample. The study included cognitively healthy older adults with MoCA scores in a narrow range, limiting the applicability of the results to clinical populations with cognitive impairments. Furthermore, the sample was predominantly female (64/87, 73.6%), raising concerns about potential gender bias in the AI model’s performance [49]. The test-retest reliability analysis was also based on a relatively small subset (n=66), which warrants caution in the interpretation of ICC values until replicated with a larger sample.

Second, the scope of our validation methodology was limited. The model’s performance was evaluated using internal cross-validation; the lack of an external validation with an independent dataset means that the model’s generalizability remains to be confirmed. Our psychometric evaluation also focused primarily on correlational and reliability metrics. Other key psychometric properties, such as responsiveness to change, were also not assessed.

Finally, it is important to acknowledge a conceptual limitation regarding what is being measured. The facial and behavioral metrics captured by the AI-CDVT, such as eye closure, are well-established proxies for vigilance. However, they are not direct measures of underlying cognitive processes. Therefore, the observed correlations with cognitive tests should be interpreted as a relationship between observable behaviors and cognitive performance, not as evidence that the AI-CDVT measures cognition in the same way as traditional tests.

### Future Directions

Building on these findings, future research should prioritize several key areas. First, the AI-CDVT must be validated in a larger, more diverse cohort, specifically including a more balanced gender representation and a wider range of cognitive abilities, to address the limitations of our current sample. Second, external validation on an independent dataset is essential to confirm the model’s generalizability. Third, its utility in clinical populations, such as individuals with mild cognitive impairment or dementia, should be explored to determine its diagnostic and monitoring potential. Finally, longitudinal studies are needed to assess other key psychometric properties, such as responsiveness to change over time or in response to an intervention.

### Conclusions

The objective of this study was to address a key psychometric weakness in a standard test of sustained attention by transforming it from a unimodal to a multimodal assessment. We demonstrated that using accessible AI tools to integrate objective behavioral data with traditional performance metrics, and we developed an AI-CDVT with superior test-retest reliability compared to the original version, while maintaining its convergent validity. The primary contribution of this work is not the development of a novel algorithm, but the demonstration that the psychometric robustness of established clinical tools can significantly be enhanced through this multimodal approach. The resulting AI-CDVT represents a more stable and reliable instrument for assessing sustained attention in older adults.

## Supplementary material

10.2196/73038 Multimedia Appendix 1 Participant demographic questionnaire and assessment version citations.

## Abbreviations

AI: artificial intelligence
AI-CDVT: artificial intelligence–based Computerized Digit Vigilance Test
CDVT: Computerized Digit Vigilance Test
CTT: Color Trails Test
ICC: intraclass correlation coefficient
MoCA: Montreal Cognitive Assessment
SCW: Stroop Color Word Test
XGBoost: Extreme Gradient Boosting
