# Supplementary material for: Artificial Intelligence–Based Computerized Digit Vigilance Test in Community-Dwelling Older Adults: Development and Validation Study
Source: JMIR Med Inform. 2025 Nov 26;13:e73038. doi: 10.2196/73038 (PMC12670460; doi:10.2196/73038)
Supplement: Multimedia Appendix 1 [file medinform-v13-e73038-s001.docx]

Appendix 1: Participant Demographic Questionnaire and Assessment Version Citations

**Part 1: Participant Basic Information**

| **Item** | **Response** |
| --- | --- |
| Participant ID | ___________ |
| Gender | □ Male  □ Female |
| Age | ___________ years old |
| Education Level | □ Illiterate □ Elementary □ Junior High □ Senior High / Vocational □ College / Equivalent □ Graduate |
| Past Medical History | □ Diabetes □ Hypertension □ Chronic Obstructive Pulmonary Disease (COPD) □ Hyperlipidemia □ Others: ___________ |
| Researcher’s Signature: __________________ | Principal Investigator’s Signature: __________________ |

**Part 2: Citations and Access Information for Copyrighted Assessment Versions Used**

As noted in our response, the following copyrighted assessments (SCW, CTT, MoCA) cannot be reproduced here. The specific versions, norms, and access details are as follows:

1. **Stroop Color Word Test (SCW) Version:**
   - Fan, W. C. (2013). *Normative Study of the Stroop Color and Word Test in Healthy Individuals in Taiwan* [Master's thesis]. Airiti Library. https://doi.org/10.6342/NTU.2013.10581
2. **Color Trails Test (CTT) Version:**
   - Ssu-Ching Cheng, Mau-Sun Hua, Yu-Chi Liao, & Hsin-Te Chang (2024). Psychometric Properties and Norms of the Trail Making Test and the Color Trails Test for Taiwan's Elderly Population: A Preliminary Study. *Chinese Journal of Psychology*, *66*(2), 215-245. https://doi.org/10.6129/CJP.202406_66(2).0002
3. **Montreal Cognitive Assessment (MoCA):**
   - The MoCA is a copyrighted instrument. It is available for download from the official website (mocacognition.com), but its use is restricted to individuals who have completed the necessary training and certification program, as specified by the test developers.
